# Epigenetic modifications in solid tumor metastasis in people of African ancestry

**DOI:** 10.3389/fonc.2024.1325614

**Published:** 2024-02-21

**Authors:** Elijah Kolawole Oladipo, Seun Elijah Olufemi, Daniel Adewole Adediran, Isaac Oluseun Adejumo, Esther Moderayo Jimah, Julius Kola Oloke, Chinedum C. Udekwu, Olorunseun O. Ogunwobi

**Affiliations:** ^1^ Genomics Unit, Helix Biogen Institute, Ogbomoso, Oyo, Nigeria; ^2^ Laboratory of Molecular Biology, Immunology and Bioinformatics, Adeleke University, Ede, Osun State, Nigeria; ^3^ Department of Biochemistry, Ladoke Akintola University of Technology, Ogbomoso, Oyo, Nigeria; ^4^ Department of Natural Sciences, Precious Cornerstone University, Ibadan, Nigeria; ^5^ Department of Biochemistry and Molecular Biology, Michigan State University, East Lansing, MI, United States

**Keywords:** African ancestry, cancer, DNA methylation, epigenetics, histone modifications, solid tumor

## Abstract

This review focuses on the critical role of epigenetic modifications in solid tumor metastasis, particularly in people of African ancestry. Epigenetic alterations, such as DNA methylation, histone modifications, alterations in non-coding RNAs, and mRNA methylation, significantly influence gene expression, contributing to cancer development and progression. Despite the primary focus on populations of European, American, and Asian descent in most cancer research, this work emphasizes the importance of studying the unique genetic and epigenetic landscapes of African populations for a more inclusive approach in understanding and treating cancer. Insights from this review have the potential to pave the way for the development of effective, tailored treatments, and provide a richer resource for understanding cancer progression and metastasis. Specific focus was placed on the role of DNA methylation, histone modifications, non-coding RNAs, and mRNA methylation in solid tumor metastasis, including how these modifications contribute to the regulation of tumor suppressor genes and oncogenes, influence cellular pathways and signaling, and interact with the immune system. Moreover, this review elaborates on the development of epigenetic-targeted therapeutic strategies and the current advances in this field, highlighting the promising applications of these therapies in improving outcomes for African ancestry populations disproportionately affected by certain types of cancer. Nevertheless, this work acknowledges the challenges that lie ahead, particularly the under-representation of African populations in cancer genomic and epigenomic studies and the technical complications associated with detecting subtle epigenetic modifications. Emphasis is placed on the necessity for more inclusive research practices, the development of more robust and sensitive methods for detecting and interpreting epigenetic changes, and the understanding of the interplay between genetic and epigenetic variations. The review concludes with an optimistic outlook on the future of epigenetic research in People of African ancestry, urging the concerted efforts of researchers, clinicians, funding agencies, and policymakers to extend the benefits of this research to all populations.

## 1 Introduction

Solid tumor metastasis is a critical issue in cancer treatment, as it refers to the spread of cancer cells from the primary tumor site to distant organs, often culminating in a vast majority of cancer-related deaths (1). The molecular mechanisms underlying this process are complex and multifaceted, and the field of epigenetics may offer significant insights into the understanding of this process (2).

Epigenetic modifications, which involve changes in gene expression without alterations to the DNA sequence itself, this plays a pivotal role in a multitude of biological processes, which includes the development and progression of cancer (3). In cancer, the most common epigenetic mechanisms include DNA methylation, histone modifications, RNA modifications, and alterations in non-coding RNAs, all of which can drive the transition from a localized tumor to metastatic disease. Importantly, unlike genetic mutations, these modifications are potentially reversible, making them promising targets for therapeutic intervention (4).

Studying solid tumor metastasis examining with the lens of epigenetics, underscores the significance of solid tumor metastasis, especially the populations of African ancestry (5). Africa which is said to be a home to a diverse array of genetic and phenotypic profiles, which in turn, may harbor unique genetic and epigenetic landscapes and variations in respect to cancer biology (6). Furthermore, most cancer research has been conducted primarily on populations of European, American and Asian descent, potentially missing crucial insights from African and other populations (7). Therefore, this review which to examine the roles of epigenetic modifications in solid tumor metastasis within African populations, which is not only important for inclusive research and treatment approaches but this could also help uncover novel pathways and mechanisms that can be therapeutically targeted.

The investigation of epigenetic modifications in solid tumor metastasis, particularly within people of African ancestry, could also offers a rich, relatively untapped resource for improving our understanding of cancer progression and metastasis (8). Insights gleaned from these studies could serve as a pointer to pave the way towards the development of more effective, tailored treatments, transforming our ability to combat cancer.

## 2 Epigenetic mechanisms involved in solid tumor metastasis

**Figure 1:**
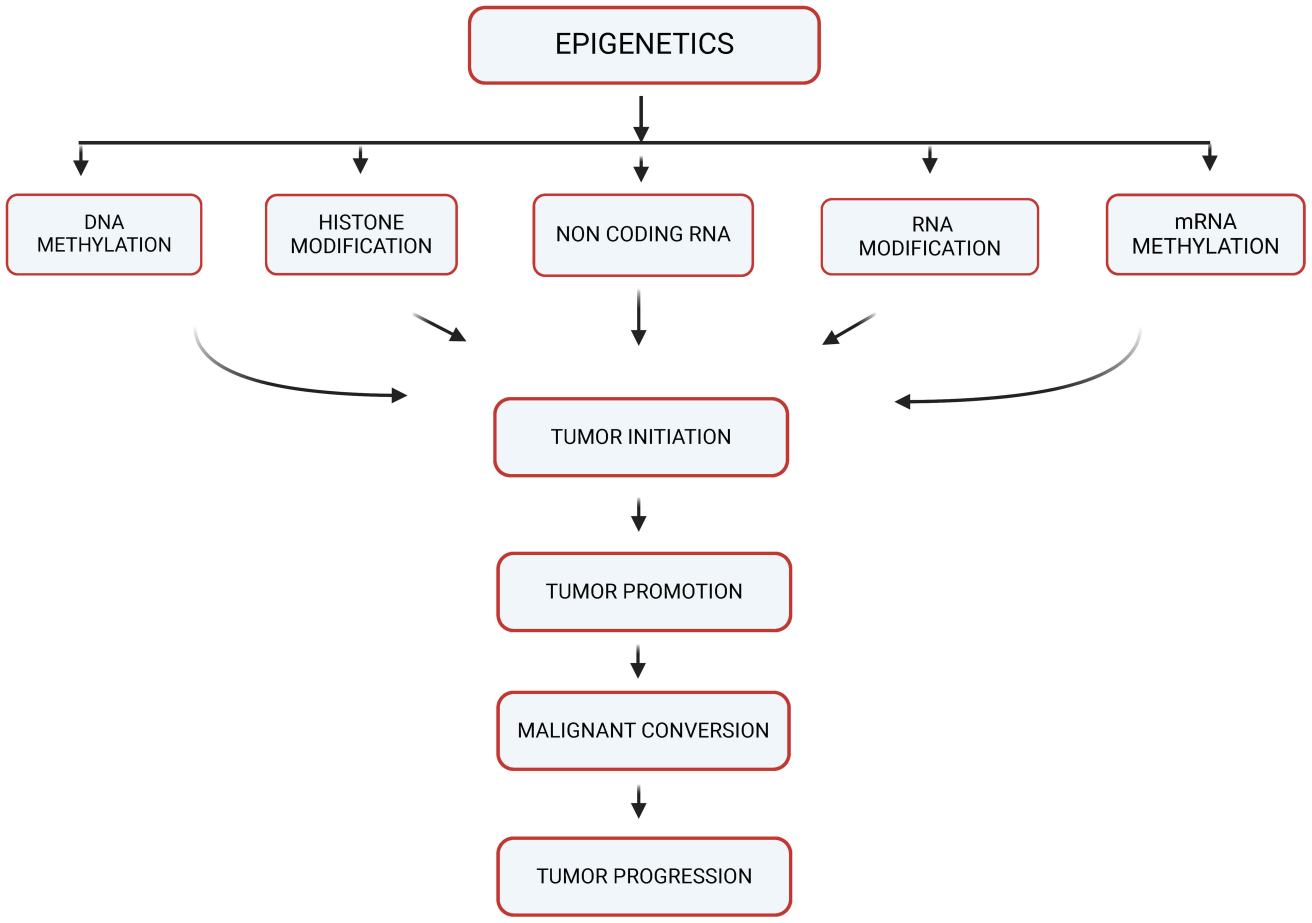
Epigenetic mechanisms potentially contributing to carcinogenesis (created with BioRender).

Epigenetics refers to changes in gene expression that don’t involve alterations to the underlying DNA sequence. Instead, these changes are caused by modification of the DNA molecule itself or the proteins (histones) around which DNA is wrapped to form a condensed structure called chromatin (9). These modifications can be stable (and thus inherited through cell division) or they may be dynamic in response to environmental signals. For solid tumor metastasis, the process by which cancer cells spread from the primary tumor to distant organs or tissues, is a complex and multifaceted phenomenon (10). It involves a series of coordinated steps that enable cancer cells to invade surrounding tissues, enter the bloodstream or lymphatic system, survive in circulation, and establish secondary tumors at distant sites (1). As shown in **Figure 1**, there is an overlap of different mechanisms of Epigenetics which include DNA methylation, Histone modification, mRNA methylation, Non-coding RNA in which emerging evidence had suggested that epigenetic mechanisms may play a critical role in regulating various aspects of solid tumor metastasis, contributing to the aggressiveness and poor prognosis of advanced cancer cases (11, 12).

### 2.1 DNA methylation and its role in gene expression

DNA methylation, one of the most studied epigenetic mechanisms, is a process that entails the addition of a methyl group to the DNA molecule, typically at cytosine residues within a cytosine-guanine (CpG) dinucleotide context (13). This modification is critical for various biological processes including embryonic development, X-chromosome inactivation, genomic imprinting, and preservation of chromosome stability. DNA methylation’s influence on gene expression is profound, with hypermethylation often associated with gene silencing, and hypomethylation linked to gene activation (14).

**Figure 2:**
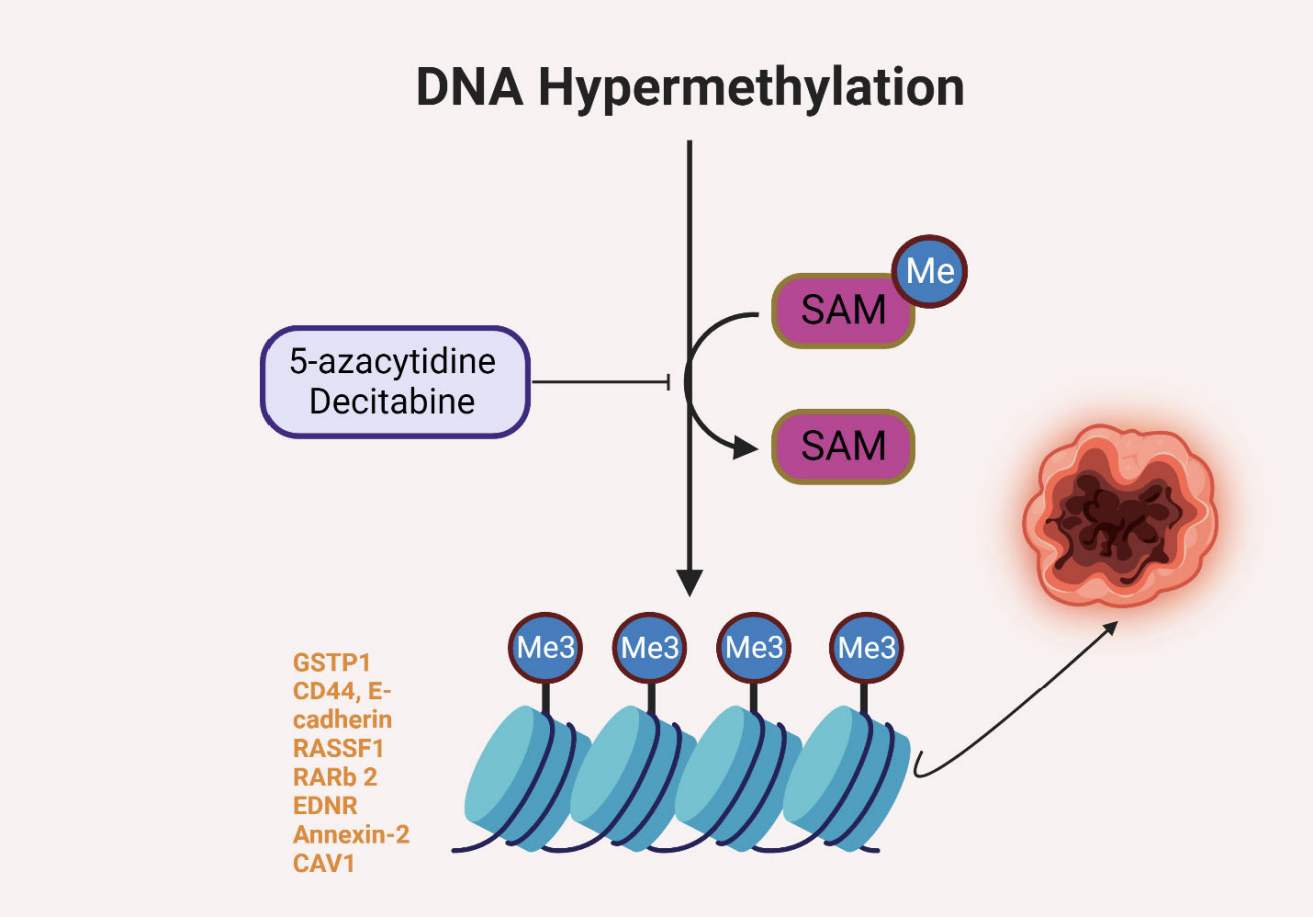
DNA hypermethylation of some genes reportedly involved in prostate carcinogenesis in men of African ancestry (15–18).

DNA methylation plays a significant role in the progression and metastasis of solid tumors. Aberrations in DNA methylation patterns, such as global hypomethylation and hypermethylation of specific gene promoters, are frequently observed in cancer (15). Hypermethylation often results in the silencing of tumor suppressor genes, contributing to uncontrolled cellular growth and proliferation, whereas hypomethylation can lead to chromosomal instability and activation of oncogenes, promoting tumor progression and metastasis (16). While there is a need for more expansive research into the specific impacts of DNA methylation on solid tumor metastasis in people of African ancestry, some studies have elucidated the role of epigenetic modifications in the progression of cancers that disproportionately affect this population group. For example, prostate cancer, which has a higher incidence and mortality rate in men of African ancestry compared to other racial/ethnic groups, has been linked to DNA methylation changes affecting several genes. These include the GSTP1, CD44, E-cadherin, RASSF1, RARb 2, EDNRB, Annexin-2, and CAV1 genes in prostate tumors, which are frequently hypermethylated, leading to their reduced expression and contributing to the aggressive nature of the disease observed in this population (17). By contrast, it was also observed that the CD44 and GSTP1 were not significantly different for methylation between populations, but there was a significantly higher methylation frequency for PYCARD (TMS1/ASC) in benign prostatic hyperplasia (BPH) for men of African ancestry (18). **Figure 2** illustrates the possible role of DNA hypermethylation in regulating expression of genes that reportedly contribute to higher incidence and worse outcomes of prostate cancer in men of African ancestry.

Given the fundamental role of DNA methylation in cancer progression and metastasis, it has been identified as a promising target for therapeutic intervention. The reversibility of epigenetic changes offers the potential for “epigenetic therapy,” which seeks to rectify the altered epigenetic states associated with cancer (18). Several such therapies are under development or in clinical use (**Table 1**), with most targeting enzymes involved in the addition or removal of DNA methylation. Among these, DNA methyltransferase inhibitors (DNMTi) such as 5-azacytidine and decitabine are used to treat hematological malignancies and have shown promise in the treatment of solid tumors (24). By inhibiting DNMTs, these drugs cause a reduction in DNA methylation, potentially leading to the reactivation of tumor suppressor genes and subsequent growth inhibition of cancer cells (25). While the potential of DNA methylation as a therapeutic target is evident, several challenges remain. One significant obstacle is the current lack of specificity of DNMT inhibitors, leading to genome-wide demethylation that can potentially reactivate oncogenes (26). Developing therapies that can specifically target the hypermethylated promoters of tumor suppressor genes remains a crucial area of research (27).

**Table 1:** Inhibitors of DNA and Histone Methylation and their applications.

Target	Inhibitor	Mechanism of Action	Examples	Clinical Application	References
DNA Methyltransferase (DNMT)	* Nucleoside analogs (5-azacytidine, decitabine) * Non-nucleoside analogs (guadecitabine) * Hydralazine derivatives * Triazine derivatives	* Covalently binds to DNMTs, leading to their degradation * Competes with S-adenosylmethionine (SAM) for binding to DNMTs * Inhibits catalytic activity of DNMTs	5-azacytidine, decitabine, guadecitabine, azacitidine, vorinostat	Myelodysplastic syndromes (MDS), Acute myeloid leukemia (AML)	Del Castillo Falconi et.al., 2022 (19)
Histone Methyltransferase (HMT)	* Small molecule inhibitors (G9a inhibitors, EZH2 inhibitors) * Peptidomimetics * Natural products (curcumin, berberine)	* Binds to the catalytic domain of HMTs, blocking substrate binding * Mimics the natural substrate, competing with histone tails for binding * Modulate HMT activity through non-specific mechanisms	SGI-110, UNC2025, BIX01294, DZNep, curcumin, berberine	Various cancers (solid tumors, hematological malignancies), neurodegenerative diseases	Zagni et al., 2013 (20), Marzochi et.al., 2023 (21) Morel et al., 2020 (22)
Lysine-specific Demethylase (LSD)	* Small molecule inhibitors (GSK2879592, IOX1) * Natural products (tranylcypromine, parnate)	* Binds to the catalytic domain of LSDs, blocking substrate binding * Inhibits catalytic activity of LSDs	GSK2879592, IOX1, tranylcypromine, parnate	Various cancers, psychiatric disorders	Hosseini and Minucci, 2017 (23)

### 2.2 Histone modifications

Histones are protein complexes around which DNA is wrapped, forming a structure known as chromatin, modifications to these histones, such as acetylation, methylation, phosphorylation, and ubiquitination, significantly influence chromatin structure and thus gene expression (28). For instance, acetylation of histones usually leads to an open chromatin structure, allowing transcriptional machinery access to the DNA, thereby promoting gene expression (29). Conversely, certain types of histone methylation can lead to a closed chromatin structure, hindering access to the DNA and suppressing gene expression (30). Histone modifications play a crucial role in the progression and metastasis of solid tumors. Aberrant patterns of these modifications can alter the expression of genes essential for controlling cell proliferation and death, ultimately contributing to tumor growth and metastasis (4) For instance, decreased acetylation or increased methylation of certain histone residues can lead to silencing of tumor suppressor genes, whereas increased acetylation or decreased methylation can result in overexpression of oncogenes.

While specific examples of genes regulated by histone modifications in solid tumor metastasis in people of African ancestry are sparse, some studies have begun to elucidate the role of these modifications in cancers disproportionately affecting this population (31). For example, in aggressive forms of prostate cancer, which is more prevalent and deadly in men of African ancestry, alterations in histone modifications have been observed. Dysregulation of histone demethylases and methyltransferases, resulting in aberrant histone methylation patterns, has been associated with increased proliferation and invasion of prostate cancer cells (32).

### 2.3 Non-coding RNA

Non-coding RNAs (ncRNAs) represent a vast class of RNA molecules that do not encode proteins but play critical roles in regulating gene expression, they comprise various subclasses, including microRNAs (miRNAs), long non-coding RNAs (lncRNAs), and circular RNAs (circRNAs), each with unique functions in cellular biology (33). For instance, miRNAs post-transcriptionally regulate gene expression by targeting mRNAs for degradation or inhibiting their translation. In contrast, lncRNAs can modulate gene expression at various levels, from chromatin remodeling to mRNA stability, while circRNAs often act as molecular sponges, sequestering miRNAs to regulate their function (34). Non-coding RNAs have emerged as key players in the epigenetic regulation of cancer progression and metastasis. For instance, some miRNAs can act as tumor suppressors or oncogenes, regulating the expression of genes involved in cell proliferation, apoptosis, angiogenesis, and other processes critical for tumor growth and metastasis (2). On the other hand, lncRNAs and circRNAs can interact with chromatin-modifying enzymes or miRNAs, influencing chromatin structure and gene expression patterns. Aberrant expression or function of these ncRNAs can thus contribute to the onset and progression of metastasis in solid tumors (35).

Comprehensive studies detailing the involvement of ncRNAs in solid tumor metastasis specifically in people of African ancestry are currently limited, certain ncRNAs have been implicated in cancers that disproportionately affect this population. For instance, in prostate cancer, a disease with notably higher incidence and mortality rates among men of African descent, several miRNAs have been reported to be differentially expressed compared to other populations, influencing tumor aggressiveness and patient outcomes (36). Similarly, a growing body of evidence suggests that lncRNAs may also play a critical role in the racial disparities observed in cancer incidence and outcomes (18). For instance, lncRNA PCA3 has been implicated in prostate cancer, with its levels reported to be significantly higher in men of African ancestry, potentially contributing to the disease’s aggressiveness in this population (37).

### 2.4 mRNA methylation

mRNA methylation, specifically N6-methyladenosine (m6A) methylation, is an important post-transcriptional modification of RNA that affects various aspects of mRNA metabolism, including stability, translation efficiency, and splicing (38). Methylation of mRNA can modulate the expression of genes without altering the DNA sequence itself, thus representing an epigenetic mechanism of gene regulation (39). mRNA methylation has been implicated in the progression and metastasis of solid tumors, aberrations in the enzymes that add the methyl mark (writers), enzymes that remove the methyl mark (erasers), and proteins that recognize and bind to the methyl mark (readers) of m6A methylation can lead to dysregulated gene expression that promotes cancer progression (40). As an instance, altered m6A methylation can affect the stability and translation of mRNAs encoding oncogenes or tumor suppressors, thus contributing to uncontrolled cell growth, immune evasion, angiogenesis, and other processes essential for tumor progression and metastasis (41). Although studies which highlights specific examples of genes regulated by mRNA methylation in solid tumor metastasis within People of African ancestry are limited, some studies have begun to elucidate the role of m6A methylation in cancers that disproportionately affect these populations. For instance, in prostate cancer, which is more prevalent and deadly in men of African ancestry, alterations in m6A methylation and its regulatory enzymes have been reported. These changes can influence the expression of genes critical for cancer progression and metastasis, potentially contributing to the aggressive nature of the disease observed in this population (42, 43).

## 3 The impact of epigenetic modifications on solid tumor metastasis in people of African ancestry on some cellular machineries

### 3.1 Tumor suppressor genes

Epigenetic modifications play a critical role in the regulation of gene expression and, consequently, in the development and progression of various diseases, including cancer. One of the most critical aspects of this process in cancer biology is the epigenetic inactivation of tumor suppressor genes.

Tumor suppressor genes play crucial roles in controlling cell growth and preventing the development of cancer (44). However, these genes can become silenced or inactivated by various epigenetic modifications, leading to uncontrolled cell growth and the development of cancer.

DNA methylation and histone modifications are two primary mechanisms by which tumor suppressor genes can be epigenetically silenced. DNA methylation often occurs at CpG islands within the promoter regions of tumor suppressor genes, preventing transcriptional machinery from accessing the DNA and leading to gene silencing (45). Similarly, certain histone modifications can alter the chromatin structure and inhibit gene expression. Conversely, non-coding RNAs and mRNA methylation can also affect the expression of tumor suppressor genes indirectly by influencing the mRNA stability and translation (46).

### 3.2 Oncogenes

Epigenetic modifications play a critical role in the activation of oncogenes, genes that have the potential to cause cancer when mutated or expressed at high levels. These modifications can lead to changes in the chromatin structure and accessibility of the DNA, ultimately impacting the transcription and expression of oncogenes (47). Several key epigenetic modifications can contribute to oncogene activation. Hypomethylation of DNA at the promoter regions of oncogenes can facilitate the binding of transcription factors and increase gene expression (48). Similarly, specific histone modifications, such as acetylation, can lead to a more open chromatin structure, promoting transcriptional activation. Other modifications, like changes in non-coding RNAs or mRNA methylation, can also influence the stability, translation, and ultimately the expression of oncogenes (49).

While the specific influence of epigenetic modifications on oncogenes in People of African ancestry is an area that warrants further research, some studies have begun to shed light on this aspect in cancers that disproportionately affect this population group. For instance, in prostate cancer, which shows higher incidence and mortality rates in African ancestry men, CaP-related oncogenic lncRNAs, such as *PVT1*, *PCAT1* and *PCAT10/CTBP1-AS*, were found to be more highly expressed in Men of Africa ancestry while compared with their counterpart of the European Ancestry (48). Epigenetic changes, such as alterations in DNA methylation patterns and histone modifications, can influence the expression of MYC, contributing to the aggressive nature of the disease observed in this population. Similarly, the epigenetic regulation of the RAS oncogene, often implicated in various cancers, might also differ among populations, potentially contributing to the observed disparities in cancer incidence and outcomes (47).

### 3.3 Cellular pathways and signaling

Epigenetic modifications play an essential role in regulating cellular pathways and signaling networks involved in the development and progression of solid tumors. By altering the expression of key genes within these pathways, epigenetic changes can influence numerous cellular processes that contribute to metastasis, including cell proliferation, survival, angiogenesis, invasion, and immune evasion (50). DNA methylation and histone modifications can result in the silencing of tumor suppressor genes or the activation of oncogenes, leading to the dysregulation of key cellular pathways (51). For instance, hypermethylation of promoter regions can inhibit the expression of genes involved in DNA repair, cell cycle regulation, and apoptosis, while hypomethylation can lead to the overexpression of genes promoting cell growth and invasion (52). Additionally, non-coding RNAs, such as miRNAs and lncRNAs, can also modulate gene expression at the post-transcriptional level, influencing cellular signaling pathways involved in metastasis (53).

Although, research is currently ongoing, some studies have begun to elucidate the impact of epigenetic modifications on cellular pathways in cancers that disproportionately affect People of African ancestry. For instance, in prostate cancer, which is more aggressive and deadly in men of African descent, analysis of data sets from The Cancer Genome Atlas (TCGA) and Genomics Resource Information Database (GRID) shows significant discrepancies in methylation patterns of African Americans versus their Caucasian counterparts (54). It has been shown that Aberrant epigenetic alterations coordinate metastatic activities across the PI3K/AKT, MAPK and Wnt/β-catenin pathways. Hypermethylation-induced PTEN silencing in the PI3K/AKT pathway which upsets the balance of signals and promotes unchecked cell proliferation. This dysregulation may be further amplified by altered histone alterations, which promote the invasion of cancer cells. The MAPK pathway undergoes DNA hypermethylation and histone modification-induced gene silence, which encourages unchecked growth and amplifies the possibility of metastatic spread. DNA hypermethylation also silences inhibitors in the Wnt/β-catenin pathway, maintaining system activity. Changes in histone modifications impact the transcription of genes, whereas dysregulated non-coding RNAs modify essential elements. A common mechanism underlying metastatic phenotypes is highlighted by the convergence of aberrant epigenetic alterations in multiple pathways, underscoring the necessity of focused therapeutic approaches that address this complex interplay (54). These pathways play critical roles in cell proliferation, survival, and metastasis. Moreover, alterations in the expression of miRNAs and lncRNAs, potentially driven by epigenetic changes, can also influence these and other signaling pathways, contributing to the aggressive nature of prostate and other cancers in People of African ancestry.

### 3.4 Immune system interactions

The interactions between tumors and the immune system, impacting the tumor microenvironment and influencing tumor progression and metastasis can be affected by epigenetics. By altering the expression of genes involved in immune response, these modifications can affect immune surveillance, evasion, and the overall immunogenicity of tumors. For instance, Resistance of melanoma to immunotherapy by immune checkpoint inhibitors is associated with global hypermethylation and low PD-L1 expression whereas global hypomethylation is associated with constitutive PD-L1 expression and inhibitory cytokine production and Hypermethylation of DNA in melanoma is associated with overexpression of DNA methyltransferases (DNMTs) and histone methyltransferase-EZH2 in the PRC2 repressive complex (55). Aberrant DNA methylation can lead to the silencing of tumor-associated antigens, reducing their presentation on the cell surface and aiding immune evasion. Similarly, changes in histone modifications can influence the transcription of genes involved in immune regulation, affecting the activity of immune cells within the tumor microenvironment (56). Also, non-coding RNAs, such as miRNAs and lncRNAs, can also impact immune responses by regulating the expression of genes involved in immune cell differentiation, activation, and function.

While comprehensive studies examining the influence of epigenetic modifications on immune system interactions specifically in people of African ancestry are currently limited, certain findings suggest a potential impact of these modifications in cancers that disproportionately affect this population (57, 58). For instance, in prostate cancer, which shows higher incidence and mortality rates in African ancestry men, alterations in the expression of miRNAs and genes regulated by DNA methylation have been linked to changes in immune responses. Such modifications can influence the immune cell composition in the tumor microenvironment, potentially contributing to the aggressive nature of the disease observed in this population.

## 4 Therapeutic strategies targeting epigenetic modifications

Epigenetic-targeted agents are designed to specifically modulate the enzymes responsible for adding, removing, or reading epigenetic marks such as DNA methylation and histone modifications. These include DNA methyltransferase inhibitors (DNMTis), histone deacetylase inhibitors (HDACis), and histone methyltransferase inhibitors (HMTis), among others (59). In addition, RNA-targeted therapeutics are being developed to modulate the levels or functions of non-coding RNAs associated with cancer progression and metastasis (60). Epigenetic-targeted agents have demonstrated clinical activities in hematological malignancies and have shown therapeutic potential in solid tumors (61). The most notable advances are seen with DNMTis and HDACis, some of which have already been approved by the FDA for specific malignancies. In solid tumors, these epigenetic-targeted agents are primarily used in combination with other therapeutic strategies, such as chemotherapy, targeted therapy, or immunotherapy, to enhance their efficacy (62). Moreover, several novel epigenetic monotherapies and combination therapies are currently under clinical investigation for solid tumors, which shows the growing interest in this field.

## 5 Future directions

Recent advances in high-throughput sequencing technologies and computational methods have revolutionized epigenetic research, offering unprecedented insights into the complexities of the epigenome and its role in disease development and progression. Single-cell epigenomic technologies are emerging as powerful tools for delineating the heterogeneity of both tumor and immune cells in the tumor microenvironment. Moreover, advances in integrative multi-omics approaches will enable researchers to simultaneously analyze genomic, transcriptomic, and epigenomic data, thus offering a more comprehensive picture of the molecular mechanisms underlying cancer progression and metastasis. Despite these advancements, there are considerable challenges in studying epigenetic modifications in solid tumor metastasis in people of African ancestry. One of the primary obstacles is the under-representation of African populations in cancer genomic and epigenomic studies. This under-representation hinders the generalizability of findings and limits our understanding of the distinct epigenetic alterations contributing to the disparities in cancer incidence and treatment outcomes observed in these populations. Further, while various techniques exist to study epigenetic changes, they have limitations that can complicate the interpretation of results, particularly in the context of population-based studies. For instance, the complexity and dynamic nature of the epigenome, combined with the technical challenges in detecting subtle epigenetic modifications, can complicate the identification and validation of cancer-associated epigenetic markers.

Addressing these challenges requires the adoption of more inclusive research practices and the development of more robust and sensitive methods for detecting and interpreting epigenetic changes. Efforts should be made to include diverse populations in epigenetic studies and clinical trials to capture the full spectrum of epigenetic alterations associated with cancer. Moreover, understanding the interplay between genetic and epigenetic variations is crucial to elucidate the molecular underpinnings of cancer disparities. Future research should also focus on the functional validation of identified epigenetic changes and the development of novel epigenetic-targeted therapies tailored to specific patient populations.

While the field of cancer epigenetics in people of African ancestry is fraught with challenges, it also offers promising opportunities for advancing our understanding of cancer disparities and developing more effective, personalized cancer treatments. Overcoming these challenges will require concerted efforts from researchers, clinicians, funding agencies, and policy makers to ensure that the benefits of epigenetic research extend to all populations.

## 6 Conclusion

In conclusion, the roles of epigenetic modifications in solid tumor metastasis, particularly in People of African ancestry, warrant deeper and more inclusive investigation. This review has underscored the significant impact of DNA methylation, histone modifications, non-coding RNAs, and mRNA methylation in gene expression and consequent cancer development and progression. While studies to date have been predominantly focused on European, American, and Asian populations, the diversity and unique genetic and epigenetic landscapes inherent in African populations offer an untapped resource that could provide pivotal insights into understanding and combating cancer more effectively. Our understanding of the intricate dynamics of epigenetic modifications could catalyze the development of more efficacious, tailored treatments and open new avenues for understanding cancer progression and metastasis.

## Author contributions

EO: Conceptualization, Supervision, Writing – original draft, Writing – review & editing. SO: Writing – original draft, Writing – review & editing. DA: Writing – review & editing. IA: Writing – review & editing. EJ: Writing – review & editing. JO: Writing – review & editing. CU: Writing – review & editing. OO: Conceptualization, Methodology, Project administration, Resources, Supervision, Writing – original draft, Writing – review & editing.
